# IndentAtlas: an open-source toolbox for indentation mapping analysis

**DOI:** 10.1016/j.mex.2026.103947

**Published:** 2026-05-06

**Authors:** Andre E. Vellwock, Shahrouz Amini, Chiara Micheletti

**Affiliations:** a Department of Biomaterials, Max Planck Institute of Colloids and Interfaces, Potsdam Science Park, Am Mühlenberg 1, 14476 Potsdam, Germany; b Department of Physics and Astronomy, Chalmers University of Technology, Kemigården 1, 412 96 Gothenburg, Sweden

**Keywords:** Indentation mapping, Nanoindentation, Data analysis, Mechanical characterization, Visualization, Software, Data curation, Data cleaning

## Abstract

Indentation is a widely used technique for characterizing the micro- and nanomechanical properties of engineered and biological materials. Despite its broad adoption, indentation data analysis, especially of large maps, still relies heavily on user input for artifact cleaning, data processing, and visualization; understanding the data before processing and analysis can drastically affect interpretation. In addition, published results of indentation maps are often generated using proprietary, non-open-source software, and commonly lack the capability to accurately correlate the probed regions of interest (e.g., optical images) with the plotted mechanical properties such as reduced modulus. Here, we present IndentAtlas, an open-source toolbox developed by and for the micro- and nanoindentation community, in which steps of post-mapping data analysis and visualization are combined into a single software platform. The toolbox focuses on the analysis of pre-processed data, rather than on curve fitting or raw data extraction. The main contributions of this work are as follows:

• A comprehensive toolbox that enables indentation users to better understand, clean, analyze, and visualize measurement results within a unified environment.
• A dedicated function for overlaying images of the sample with spatially resolved indentation data, such as reduced modulus and hardness.

## Graphical abstract

**Figure:**
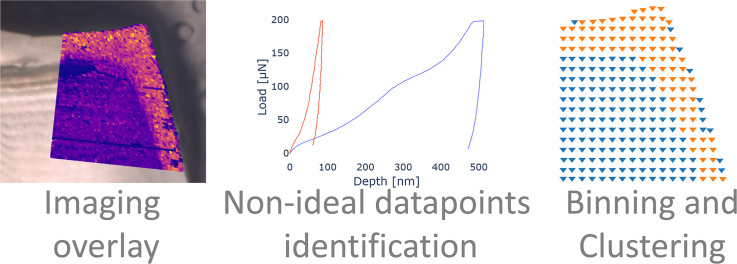

## Specifications table

**Table:** 

**Subject area**	Materials Science
**More specific subject area**	Indentation
**Name of your method**	Understanding, artifact cleaning, analyzing, and plotting of data acquired with indentation technique
**Name and reference of original method**	N.A.
**Resource availability**	Resources referred to in this article: IndentAtlas (https://github.com/chiaramicheletti/IndentAtlas.git)

## Background

Micro- and nanomechanical material properties, such as hardness and reduced modulus, are commonly measured using indentation and provide highly valuable information for materials scientists and engineers. These properties are directly linked to the underlying structure and functional performance of the probed material. With the rapid advancement of nanotechnology and semiconductor industries, the use of indentation has increased steadily over recent years [1]. As a localized and quantitative mechanical characterization technique, it enables new pathways for investigating complex materials and structure-property relationships [[2], [3], [4], [5], [6], [7]]. Indentation mapping experiments require specialized instrumentation, most often commercial triboindenters produced by established manufacturers, as custom-built systems are relatively uncommon. After acquisition, measurement data are typically exported and processed using separate software tools. Data analysis and visualization frequently rely on commercial software packages, such as Origin, which are not specifically designed for indentation applications. Consequently, their functionality for indentation-specific tasks is limited, and their use introduces additional financial barriers. To address these limitations, a few research groups have developed custom analysis scripts but often without a user-friendly interface [[8], [9], [10]]. However, these scripts are usually designed for narrow, task-specific purposes, such as data cleaning, statistical analysis, or plotting, and rarely provide an integrated workflow. Furthermore, many scientists also perform these tasks manually [11], which can introduce errors and possible biases. As a result, indentation data processing often remains fragmented across multiple tools, increasing user effort and reducing reproducibility. These challenges highlight the need for a multi-purpose open-source toolbox, i) allowing the users to visually understand the collected map data, and ii) enabling analysis of pre-processed datasets from different indentation instruments, provided they are formatted into a standardized input structure. The goal of this work is to introduce IndentAtlas, a toolbox designed to facilitate the complete indentation data workflow. IndentAtlas compiles essential functions within a single environment, assisting users in cleaning anomalous or artifactual load-displacement curves, performing statistical analyses, and generating spatially resolved property maps overlaid with images of the sample. Despite not providing raw curve extraction, curve fitting, or area-function calibration, we anticipate that this toolbox will serve as a valuable resource for the indentation community.

## Method details

**Fig. 1:**
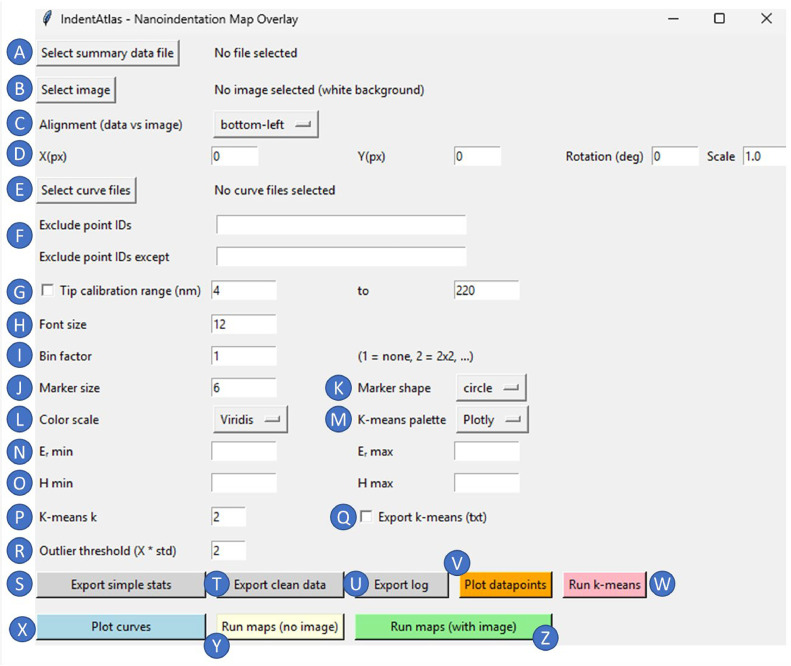
Graphical user interface (GUI) of the IndentAtlas software. The interface provides access to all major functions, including data and image import, alignment and scaling, curve filtering based on tip calibration range, outlier detection, spatial binning, clustering analysis, visualization options, and data export. Individual options are labelled alphabetically (A–Z) and are referenced throughout the manuscript for clarity.

The source code is available at GitHub (https://github.com/chiaramicheletti/IndentAtlas.git). The software was developed in Python and further refined and optimized using large language models (ChatGPT 5.1, OpenAI). Data visualization was performed using Plotly, and numerical analysis used NumPy, Pandas, and scikit-learn. In this section, we focus on describing the practical usage of the software. After opening and running the Python script, the graphical user interface (GUI) appears after a few seconds (Fig. 1). The interface serves as the primary point of interaction with the software. The available options and their functions are described in detail below.

(A) Select the *.txt summary file, previously exported from the indentation equipment (i.e., after post curve-fitting, summarizing reduced modulus, hardness, etc., for each load-displacement curve), such as triboindenters from Bruker/Hysitron. The summary *.txt file should be pre-prepared, containing all data of the acquired datapoints. If you have custom data, please use the *.xlsx example file (see GitHub) and format your data accordingly. Please do not modify the first row in the *.xlsx file. Currently, IndentAtlas does not natively support the import of proprietary data formats from different manufacturers.
(B) Select the image of the sample. It can be any image acquired with any technique (e.g., light microscopy or scanning electron microscopy). This is optional, if no image is selected, white will be the standard background.
(C) Select what the alignment between the image and datapoints is. These refer to where the first datapoint was acquired in relation to the center of the sample image.
(D) If necessary, adjust the alignment manually using x- or y-axis corrections, or rotation and scale adjustments.
(E) Select multiple *.txt files with the curves data, such as files exported by triboindenters from Bruker/Hysitron. If you have data from other equipment, please set depth and load in the first and second column of each *.txt file, respectively (see example files in the GitHub for necessary formatting). This step is optional.
(F) Select the point ID(s) that you would like to remove from the analysis and plotting. Alternatively, you can specify the ID(s) of the points to keep in the other field. It accepts inputs such as “1,2,3”, thus removing/keeping point IDs 1, 2, and 3; and “1-100”, thus removing/keeping all points between 1 and 100.
(G) If ticked, only datapoints within the tip calibration range (set by the user) will be considered for analysis and plotting.
(H) Font size for all the graphs.
(I) Bin factor, where neighboring datapoints can be averaged into one.
(J) Datapoint marker size in the map plotting.
(K) Datapoint marker shape in the map plotting, e.g., conospherical (circle) and Berkovich tips (triangle-down) can be represented.
(L) Color scale in the map plotting.
(M) Color scale in the k-means clustering map plotting.
(N) Maximum and minimum values of reduced modulus to be considered.
(O) Maximum and minimum values of hardness to be considered.
(P) The number of clusters to be considered for the k-means clustering.
(Q) If ticked, it will export a *.txt file with the basic statistics of the k-means clustering.
(R) The threshold to flag datapoints as outliers when the user selects the “Plot Datapoints” function. The value entered defines how many standard deviations (*σ*) from the mean of the selected variable (i.e., either reduced modulus or hardness) a datapoint must lie to be considered an outlier. The method is applied globally to the dataset and highlights potential outliers without automatically removing them. This approach may not be appropriate for multimodal datasets, where datapoints belonging to distinct material phases may be flagged as outliers; therefore, interpretation should be performed with care.
(S) A *.txt file is generated with common statistics (mean, minimum and maximum values, etc.).
(T) A *.txt file is generated for the cleaned data (e.g., after removing user-selected datapoints).
(U) A *.txt file is generated with all the user input in the GUI.
(V) Graphs for reduced modulus, hardness, and contact depth are plotted, showing the distribution of datapoints and outliers.
(W) Graphs for k-means clustering are plotted based on a selected variable (either reduced modulus or hardness). For each variable, it includes elbow and silhouette plots, scattering with box plots of datapoints, and map plots. Box plots represent the median, interquartile range, and whiskers extending to 1.5x the interquartile range. The elbow method evaluates the within-cluster variance, while the silhouette score quantifies cluster separation and cohesion. While the elbow method is commonly used as a heuristic for selecting the number of clusters, it should be interpreted with caution. Recent studies have highlighted that the elbow criterion can be ambiguous and may not reliably indicate an optimal number of clusters, particularly for complex or noisy datasets [12]. Therefore, in this work, the elbow plot is used only as a qualitative guide and is complemented by additional metrics such as the silhouette score, as well as user interpretation.
(X) All the load vs. displacement curves are plotted.
(Y) Map plots for reduced modulus and hardness are plotted, without background image.
(Z) Map plots for reduced modulus and hardness are plotted, with background image.

## Method validation

The performance and utility of the software were demonstrated through three representative case studies, described in detail below. A study using an earlier version of the software has already been published [13].

Example 1 ***Longitudinal section of a mantis shrimp raptorial appendage***

**Fig. 2:**
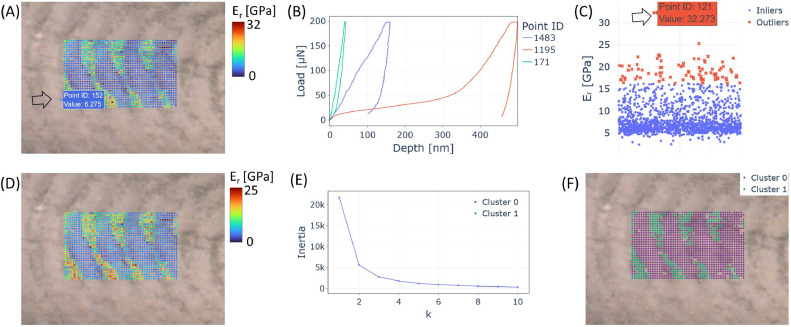
Longitudinal section of the mantis shrimp raptorial appendage. (A) Overlay of reduced modulus map with the optical image and identification of a misplaced datapoint. (B) Example of load-displacement curves, showing out-of-calibration examples. (C) Outlier datapoints identification. (D) Cleaned map after removal of selected points. (E) Elbow plot aiding the selection of the number of clusters to use in k-means clustering. (F) Map showing the clusters and their locations.

In the first example (Fig. 2**A–F**), we analyzed a longitudinal section of the stomatopod raptorial appendage of a mantis shrimp [14]. The sample appendage was removed and cold-embedded in acrylic resin, followed by sectioning and step-wise polishing. Detailed methodology of sample preparation is described elsewhere [14]. Indentation data were acquired using a triboindenter TI-950 (Hysitron, Minneapolis, USA) with a conospherical tip (1 µm radius) and 2 µm lateral spacing. After processing the curves using Oliver-Pharr model [15], data were exported in *.txt format.

After opening IndentAtlas, by selecting the indentation data file in *.txt format using Option A (Fig. 1) and the corresponding optical image using Option B (Fig. 1), the user can generate an overlaid visualization of the two datasets (Option Z, Fig. 1) and subsequently obtain an overlaid image (Fig. 2**A**). In this case, the alignment between the optical image and the indentation grid was defined at the bottom-left (Option C, Fig. 1), meaning that the bottom-left corner of the datapoint array is positioned at the center of the optical image. The initial map (Fig. 2**A**) reveals regions of high reduced modulus (i.e., >10 GPa) intercalated with lower-modulus regions (i.e., <10 GPa), a characteristic feature of the biological composite. Several incorrectly positioned datapoints are also visible, such as point ID 152. Following, we proceeded with two cleaning approaches. First, after importing selected load-displacement curves using Option E (Fig. 1) and visualizing the curves with Option X (Fig. 1), see Fig. 2**B**, we identified curves with large contact depths. Here we decided to import only selected curves, rather than all acquired datapoints, for easier visualization. Second, considering that the indenter tip was calibrated for contact depths between 4 and 220 nm, a substantial number of measurements were found to exceed this calibration range. While such curves are not necessarily invalid, they can only be reliably interpreted if additional tip calibration is performed for the relevant depth range. In the present analysis, datapoints outside the calibration limits were therefore excluded using Option G (Fig. 1). Subsequent plotting of the remaining datapoints using Option V (Fig. 1) revealed several statistical outliers (highlighted in red in Fig. 2**C**), which is expected given the pronounced material heterogeneity of the sample. However, certain points, such as point ID 121, were identified as excessively extreme. This interpretation relies on user knowledge, but it can be automated by removing every datapoint above the chosen deviation. These points were manually selected for removal using Option F (Fig. 1), after which the reduced modulus map was replotted, resulting in a substantially cleaner spatial distribution (Fig. 2**D**). This further illustrates how our software can aid data analysis and interpretation. By plotting spatially resolved data alongside load-displacement curves and statistical visualization (jitter plots), measured mechanical properties can be more readily interpreted, while iterative repetition of the plotting process, e.g., by progressively excluding anomalous indentation curves and outliers, allows further refinement of the analysis.

For layered materials with strongly contrasting mechanical properties, clustering approaches can provide additional insights. Using k-means clustering, we first set it to two clusters in Option P (Fig. 1) and plot via Option W (Fig. 1). The elbow plot (Fig. 2**E**) clearly shows a noticeable change in slope around k = 2. However, given the known limitations of the elbow method [12], this result is interpreted in conjunction with the silhouette score and the spatial distribution of clusters, showing a clear separation of two distinct clusters (Fig. 2**F**) that matches the observed high- and low-reduced modulus regions. The clustered results can be exported as a *.txt file using Option Q (Fig. 1). To facilitate further replication, we can also export the log of user inputs with Option U (Fig. 1).

Example 2 ***Transversal section of a desert locust digging valve***

**Fig. 3:**
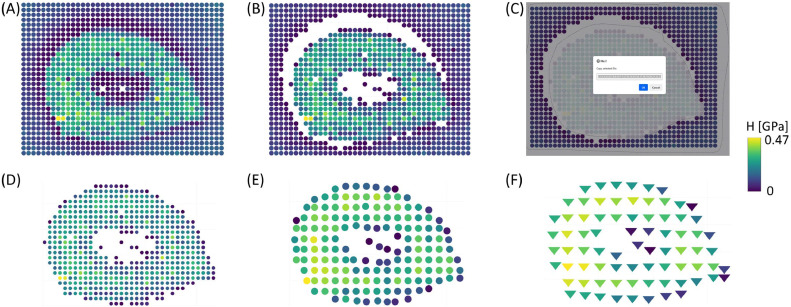
Validation with a transversal section of a desert locust digging valve embedded in epoxy resin. (A) Pre-processing result of hardness measurements. (B) Removal of measurements outside of the tip calibration. (C) Isolation of sample-only data and further (D) map plot. (E-F) The effect of spatial binning on map visualization, and (F) different marker shapes.

In the second example (Fig. 3**A-F**), a transversal section of the digging valve of a desert locust was analyzed [16]. One valve was removed and embedded in epoxy resin, followed by sectioning and step-wise polishing, showing a transversal section. Detailed methodology of sample preparation is described elsewhere [16]. Indentation data were acquired using a triboindenter TI-950 (Hysitron, USA) with a conospherical tip (1 µm radius) and 10 µm lateral spacing. After processing the curves using Oliver-Pharr model [15], data were exported in *.txt format.

In our proposed toolbox, after importing the indentation dataset using Option A (Fig. 1), we can already plot a map of hardness values (Fig. 3**A**), using Option Y (Fig. 1). Following the removal of datapoints outside the tip calibration range using Option G (Fig. 1), it becomes evident that most excluded measurements are located along the sample-epoxy interface (Fig. 3**B**). This behavior is commonly observed when partial detachment occurs between the sample and the embedding medium during preparation, leading to anomalously high contact depths. A similar effect is observed in the central region of the sample, because it is biologically hollow (known as lumen). The software effectively highlights both scenarios, facilitating efficient identification and removal of unreliable measurements. By manually selecting datapoints located outside the sample boundaries (Fig. 3**C**), with either box or lasso selection options, the user can copy the datapoints and remove them using Option F (Fig. 1), thus generating a clean mechanical property map of the biological structure (Fig. 3**D**). To further simplify the dataset, spatial binning can be applied using Option I (Fig. 1). It is essential to highlight that binning can mask important features, thus requires careful consideration before its application. Binning by a factor of two or even three results in coarser, yet smoother, representations of the reduced modulus distribution (Fig. 3**E** and Fig. 3**F**, respectively). In the latter (Fig. 3F), we demonstrate the “triangle-down” marker shape, representing Berkovich indents.

Example 3 ***Commercially pure titanium***

**Fig. 4:**
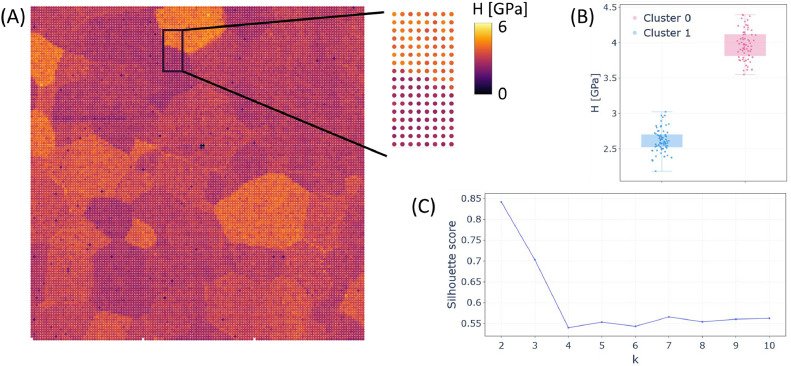
Validation using a nanoindentation dataset from commercially pure titanium. (A) Hardness map highlighting the region selected for detailed analysis. (B) K-means clustering (k = 2) separating two hardness populations within the selected region, and distribution of hardness values for each cluster presented as scatter and box plots. Box plots represent the median (central line), interquartile range (box), and whiskers extending to 1.5x the interquartile range. (C) Silhouette score as a function of the number of clusters (k), indicating that two clusters provide a clear separation of the data.

In the third example (Fig. 4**A–C**), we use a dataset acquired with a G200 TriboIndenter (Agilent Technologies/KLA Tencor) [17], in which commercially pure titanium samples were embedded and subsequently polished stepwise. The detailed sample preparation methodology is described elsewhere [17]. The dataset is particularly useful because it was obtained using different equipment than in the previous examples, and the original output format is *.mat. Therefore, the file was first converted and reformatted to match the *.xlsx example file structure, following the same column order (see GitHub). The dataset was then imported using Option A (Fig. 1), and the hardness map was generated using Option Y (Fig. 1). The resulting map (Fig. 4**A**) clearly reveals the material grains and their interfaces. To further investigate these interfaces, a smaller region was selected using Option F (Fig. 1) (see the selected region in Fig. 4**A**), and k-means clustering was applied (Fig. 4**B**). Using two clusters appears appropriate, as indicated by a high silhouette score, suggesting good separation between clusters. This example further illustrates that IndentAtlas can be used with input data from different manufacturers of triboindenter.. The purpose of this example is to demonstrate the application of the toolbox to a realistic experimental dataset, rather than to validate the measured mechanical properties against reference values.

### Limitations

IndentAtlas depends on the quality and format of the indentation data input and assumes that spatial coordinates and mechanical properties are correctly exported from the instrument software or adequately formatted by the users. Filtering based on contact depth is limited to user-defined calibration ranges and does not replace proper indenter calibration. Image-to-data alignment is performed semi-manually, which provides flexibility but introduces user-dependent variability. At present, no quantitative metric (e.g., residual alignment error) is implemented to assess alignment accuracy, and therefore different users may obtain slightly different overlays. The software focuses on post-processing and visualization and does not perform raw curve fitting or mechanical property extraction from load-displacement data. While the GUI provides visual statistical summaries (e.g., box plots), further integration of advanced statistical metrics directly into the GUI will be considered in future versions. Future improvements will focus on expanding data import compatibility, reducing user-dependent alignment steps through semi-automated image registration, and incorporating more advanced statistical and clustering methods. Additional support for curve-level analysis and calibration-aware filtering may further extend the applicability of the software to a broader range of experimental conditions. The current implementation has been validated on multiple experimental datasets, but does not yet include a formal unit testing framework or synthetic validation datasets.

## CRediT author statement

**Andre E. Vellwock**: Software, Validation, Formal analysis, Writing - Original Draft, Visualization; **Shahrouz Amini**: Resources, Writing - Review & Editing, Supervision; **Chiara Micheletti**: Conceptualization, Software, Formal analysis, Writing - Review & Editing.

## Related research article

None.

## For a published article

None.

## Declaration of competing interest

The authors declare that they have no known competing financial interests or personal relationships that could have appeared to influence the work reported in this paper.

## Data Availability

Data will be made available on request.
